# Patients’ and next of kin’s expectations and experiences of a mobile integrated care model with a home health care physician – a qualitative thematic study

**DOI:** 10.1186/s12913-023-09932-4

**Published:** 2023-08-29

**Authors:** Lina Emmesjö, Catharina Gillsjö, Anna K. Dahl Aslan, Jenny Hallgren

**Affiliations:** 1 School of Health Sciences, University of Skövde, P.O. Box 408, SE-541 28 Skövde, Sweden; 2 School of Health and Welfare, Jönköping University, Jönköping, Sweden; 3 College of Nursing, University of Rhode Island, Kingston, RI USA

**Keywords:** Patient, Next of kin, Home health care, Home health care physician, Nursing, Municipal care, Thematic analysis, Qualitative

## Abstract

**Background:**

The organizational principle of remaining at home has offset care from the hospital to the home of the older person where care from formal and informal caregivers is needed. Globally, formal care is often organized to handle singular and sporadic health problems, leading to the need for several health care providers. The need for an integrated care model was therefore recognized by health care authorities in one county in Sweden, who created a cross-organisational integrated care model to meet these challenges. The Mobile integrated care model with a home health care physician (MICM) is a collaboration between regional and municipal health care. Descriptions of patients’ and next of kin’s experiences of integrated care is however lacking, motivating exploration.

**Method:**

A qualitative thematic study. Data collection was done before the patients met the MICM physician, and again six months later.

**Results:**

The participants expected a sense of relief when admitted to MICM, and hoped for shared responsibility, building a personal contact and continuity but experienced lack of information about what MICM was. At the follow-up interview, participants described having an easier daily life. The increased access to the health care personnel (HCP) allowed participants to let go of responsibility, and created a sense of safety through the personalised contact and continuity. However, some felt ignored and that the personnel teamed up against the patient. The MICM structure was experienced as hierarchical, which influenced the possibility to participate. However, the home visits opened up the possibility for shared decision making.

**Conclusion:**

Participants had an expectation of receiving safe and coherent health care, to share responsibility, personal contact and continuity. After six months, the participants expressed that MICM had provided an easier daily life. The direct access to HCP reduced their responsibility and they had created a personalised contact with the HCP and that the individual HCP mattered to them, which could be perceived as in line with the goals in the shift to local health care. The MICM was experienced as a hierarchic structure with impact on participation, indicating that all dimensions of person-centred care were not fulfilled.

## Background

The organizational principle of remaining at home has globally offset health care from the hospital environment to the home of the older person [1–4], which many older persons in Sweden prefer [5], and has been known to increase survival rates without increasing medical costs [6]. Older persons with multiple health problems often need health and social care from several providers [7–9], including informal care from next of kin [10, 11]. Informal care givers are often the patient’s partner or adult children [12, 13]. The support from informal care givers is often a necessity for older persons to continue living at home [14] and is motivated by their relationship with their next of kin [15]. Independent of the context, the informal care givers providing care within the home generally lack health care education as well as rarely get paid for their efforts [10, 16, 17]. Strategies have been implemented to make payments directly to informal care givers, or compensate them for potential earnings. Support for informal care givers have been instigated, such as educational programs or respite care [10]. However, caregiver burden is common among informal caregivers [18–20]. They might also face psychological stress and depression [21]. Older persons who have a next of kin involved in their care will often seek emergency health care less often, which highlights the importance of social support, as well as the next of kin’s involvement in health care [22, 23].

Globally, formal care is predominantly organized to handle singular and sporadic health problems [7, 24–27], with a fragmented organization [28, 29], where declining coverage of health care services has been found in the Nordic countries, driven by the need to prioritize recourses [30]. In Sweden, health care is tax funded, where 21 regions are responsible for hospitals and primary care. The regions are responsible to provide quality health care for those living in the region, where primary care is to ensure quality care close to the citizens. Furthermore, the region is responsible for ensuring the possibility to choose health care provider [31], which has led to an increasing number of private primary health care centers [32–34] partly undermining the integration between health care authorities [28]. Sweden also has 290 municipalities which are responsible for providing the support and help to the individual with what they need, called social care [35]. Furthermore, the municipality is responsible for providing health care for those in need of home health care up to the level of registered nurses [31]. To have access to home health care, the patient is assessed by a physician at a primary health care center before being admitted to home health care, based on the older patient’s capability of traveling to the primary health care center. The structure is dictated by the ÄDEL reform stating that the municipality is responsible for continuous health care for older persons with complex care needs, but only to the level of nurses [36]. The Swedish government has defined geriatric health care as fragmented because of having several providers, as the responsibility of providing health care is shared between the municipality, primary and private care, which has led to uncertainty about health care responsibility [29]. This fragmentation has resulted in differences in the care patient receive based on which municipality the patient is living in [30]. A structural change in Swedish health care is currently underway and has been described as a shift to local health care. The goal of local health care is to provide close, coherent, person-centered and integrated care that strengthens health for patients, allowing for patient participation and health care resources being used efficiently [37, 38]. However, so far, the changes to reach the goals of this shift to local health care are mainly on a structural level [38].

The health care authorities in Sweden have nationally expressed the need for integrated care models to provide quality health care for patients to strengthen the collaboration between the municipality and primary care to handle the shift to local health care. Furthermore, a hope is that integrated care models will solve problems to the fragmented health care system [39]. For patients, integrated health care can support patients with complex care needs [24, 40]. Integrated care brings together several professions over organizational borders [41–47] and has especially been applied to long-term care [40]. Long-term care includes a broad range of support, aiming to prevent, reduce or rehabilitate functional decline for patients in different health care settings, such as hospital or municipality care [13]. Several ways of working in integrated care, such as collaborations between municipality nurses and primary health care physicians [46], as well as family physicians working in different organizations, has been recognized throughout the world [44]. Integrated care can lead to improved health and social care [48], functional abilities, and mental well-being [46]. Specifically, integrated care has been shown to reduce unnecessary hospitalization in several countries [49–52] and provide a faster response time to patient needs, as well as more informed and improved assessments [45, 52]. However, integrated care can be difficult to implement [52–54] and is considered more positively by those who have singular health problems rather than multiple issues [55]. Patients have further described how patient participation in developing integrated care is crucial to enhancing the quality of care [56]. Integrated care and care coordination has been promoted in Sweden [28] and as being connected to the organization of the health care system. To bridge the gap between health care providers which the fragmented health care system had created, the health care authorities in one county created a cross-organizational, integrated care model. The care model means to provide coherent health care, to lower unnecessary hospitalizations, improve health care within the home, and to meet the challenges of a growing older population that is receiving health care in the home [57].

### Mobile integrated care model

The mobile integrated care model with a home health care physician (MICM) is a collaboration between regional and municipal health care authorities, and is one part of a three parted care model [58]. The care model was created by the health care authorities, including representatives from hospital, primary, and municipality health care, in 2010 in one county consisting of 15 municipalities in Sweden. The MICM was negotiated and agreed on by the health care authorities on the basis of the legal obligation of primary health care centers to provide physician care towards municipality patients [59]. The need for an integrated care model was perceived through the organizational obstacles of the fragmented health care system [36]. The focus of the MICM is health care, so those receiving care within it are referred to as patients. The main collaboration partners working towards the patient in the MICM are RN and physicians. However, collaboration can also extend to include all other personnel working in health care in the municipality, such as assistant nurses (AN), occupational therapists and physiotherapist.

Before implementing the MICM:

- There was often no prearranged physician working with home health care,
- physician visits were conducted at the primary health care center.
- The patients rarely met a physician because medical planning was done through the municipality nurse, and,
- each week, different primary health care physicians—at times, locum physicians—could be responsible for municipality health care.

The MICM includes the following:

- Having an appointed physician (MICM physician),
- the municipality registered nurse (RN) and the MICM physician making home visits to the patient, and,
- the integrated team co-creating a medical health care plan (MHCP) with the patient and their next of kin at least once a year.

The MICM was created from a person-centered care perspective [50, 57]. Person-centered care involves keeping the patients active in the planning and execution of health care and helping them gain a meaningful life beyond their diagnosis while also building a partnership with the patient [60–64]. Specifically, the structure of the MICM is promoted by person-centered care through co-creating health care plans with the health care professionals, patients, and next of kin, along with added accessibility and continuity. While aiming to be cost effective, the MICM entails providing coherent quality health care to older persons who are living at home and dealing with extensive and complex health care needs, being the main health care contact for the patient [65, 66]. In the majority of the municipalities where the MICM has been implemented, all the patients are admitted into the care model when admitted to home health care, which is dictated by the agreement made by the health care authorities which created the MICM [59]. Previous studies have described the RN and MICM physician perspectives on the MICM [67, 68], expressing the importance of building relationships between team members, as well as with the patient and next of kin. Furthermore, previous findings suggest that, although many patients and next of kin are pleased with the MICM, there are still obstacles. A review study focusing on integrated care shows that patients’ and next of kin’s experiences of integrated care are lacking [69]. To evaluate the perspective of the patient and next of kin would deepen the understanding of integrated home health care models and the possibility of enhancing care.

### Aim

To illustrate patients’ and next of kin’s expectations and experiences of the mobile integrated care model with a home health care physician at baseline and at six months of follow-up.

## Method

An inductive, qualitative study design with semi-structured interviews and field notes was used. Qualitative studies are more adaptable to illustrate multiple experiences of reality [70], which suited the aim of the study. An inductive approach moves from the specific to the general, and is useful when wanting to enhance the knowledge about a subject [71]. The qualitative approach allowed for the exposure of the participants’ expectations and experiences. Data collection was initially done before the patients met the MICM physician and then again six months later.

### Setting

The Heads of the Department of Health and Social Care from 15 municipalities were asked to consent to data collection within their organization, with 12 municipalities fulfilling the inclusion criteria of having had MICM for at least 6 months. The municipalities were chosen because they were the ones who had participated in developing the MICM and had worked with it the longest. The municipalities ranged from having 2,000 to 35,000 citizens.

#### Recruitment

Unit managers or the medically responsible RN in each municipality appointed a contact person to help identify participants who met the inclusion criteria. To be included, the patients had to have daily services from municipality home care and not yet have met the MICM physician. The next of kin was a family member of a patient who participated in the study, as chosen by the patient. The participants (patients and next of kin) had to be willing to participate, be able to understand a question, and hold a conversation in Swedish. The contact person then informed the patients both orally and in writing, through an information letter, about the study. The patients were informed that participation in the study was voluntary and that participation or lack thereof would not affect their health care. Patients who agreed chose a next of kin whom they wished to include. Patients who showed interest in participating were asked if their contact information could be given to the researchers. For patients who agreed, their contact information was delivered from the RN to the researcher over the phone. The researcher then called the patient to inform them about the study once more, and if interest in participating remained, a time and place for data collection was planned. The patient then provided contact information to the next of kin, whom the researcher then contacted to inform them about the study and ask if they wished to participate as well. Monthly meetings were held between the researchers and contact persons to discuss recruitment and avoid selection bias.

#### Participants

The plan was to recruit 50 patients and 50 next of kin to collect both qualitative and quantitative data. However, because of difficulties in recruiting participants, the number of participants was lower. Data collection was initially done with 17 patients and 17 of their next of kin and, at follow-up, with 15 of each participation group (Table 1). The quantitative data collection included the questionnaires RAND-36 and Sense of Coherence (SOC-13), which were intended for use in a mixed method study. However, because of difficulty recruiting participants, the quantitative data collection could only be used to describe the participant group (Table 1). The RAND-36 questionnaire [72] measures quality of life and self-evaluated health through a self-evaluating instrument from 0 (lowest) to 100 (highest). SOC-13 [73] measures comprehensibility, manageability, and meaningfulness in scores between 13 and 91, where 91 is the highest sense of coherence [73, 74]. The RAND-36 score among the participants was similar in physical functioning, physical and emotional role limitations, and emotional well-being to another study with a similar population and setting, while the participants in the current study had a higher score of energy/fatigue, social functioning, and general health [75]. The total SOC score in the current study was slightly higher than a similar group in a similar setting [75], which may indicate the participants self-evaluated health in relation to others in a similar context. The scores are presented both for baseline and for the follow up, to describe both the participants at baseline and after six months. It is possible that the participants self-evaluated health and sense of coherence may influence the participants expectations and experiences, however, no such analysis was made due to the low sample of participants.

**Table 1:** Participant’s characteristics and data collection execution at baseline and at follow up

	Patient initially n = 17	Patient follow up n = 15	Next of kin initially n = 17	Next of kin follow up n = 15
**Age**	72–101	72–95	33–79	33–79
**Female, n**	9	8	15	13
**Male, n**	8	7	2	2
**SOC-13**, **mean (sd)**	79.76 (7.01)	78.66 (7.41)	78.23 (6.77)	77.20 (9.68)
**Physical functioning, mean (sd)**	22.05 (17.32)	26.66 (22.80)	71.47 (29.03)	70.33 (29.54)
**Role limitations physical health, mean (sd)**	30.88 (32.51)	28.33 (29.68)	61.76 (42.49)	65.00 (42.04)
**Role limitations emotional problems, mean (sd)**	78.43 (38.98)	84.44 (33.01)	86.27 (33.45)	95.55 (11.72)
**Energy fatigue, mean (sd)**	57.72 (22.70)	56.33 (21.41)	67.05 (22.78)	64.00 (17.02)
**Emotional wellbeing, mean (sd)**	74.82 (18.64)	76.80 (20.46)	79.52 (15.02)	84.26 (11.85)
**Social functioning, mean (sd)**	71.02 (25.28)	55.83 (33.69)	80.14 (28.31)	78.33 (23.36)
**Pain, mean (sd)**	58.08 (31.30)	57.16 (34.63)	68.82 (32.23)	66.33 (34.75)
**General health, mean (sd)**	49.70 (26.12)	50.00 (31.95)	60.88 (25.99)	62.66 (22.74)
**Cohabitating partner, n**			5	5
**Adult child, n**			12	10
**Met together, n**	13	10	13	10
**Met separately, n**	4	5	4	5
**Phone meeting, n**	1	1	4	5
**In-person meeting, n**	16	14	11	9
**Video link meeting**	0	0	1	1

### Data collection

Data were collected between September 2021 and October 2022. During the first visit to the participants, the study was introduced, and oral and written informed consent was obtained. The researcher collected the participant’s demographic data before starting the audio-recorded interview. After the interviews, the participants filled out two questionnaires: a self-evaluated sense of coherence (SOC-13) [73] and health (RAND-36) [72]. The first author, who had previous experience in performing interviews, taking field notes, and working with older persons and next of kin, collected all the data. The initial interviews lasted 19 to 78 min, and the follow-up interviews lasted between 10 and 64 min. There were two different semi-structured interview guides with similar questions at each data collection. One was directed towards patients and the other one towards next of kin. The initial interview focused on the participants’ expectations of MICM. The first question for patients was, “What are your expectations of receiving home health care with a home health care physician?” The first question to next of kin was, “What are your expectations of your relative receiving home health care with a home health care physician?” The questions for both patients and next of kin in the interviews focused on support in daily life, security, sense of home and well-being. The focus of the interviews 6 months later was the experiences with the MICM, with the initial question for patients being the following: “What are your experiences of receiving home health care with a home health care physician?” The following question was asked of next of kin: “What are your experiences as a next of kin having a relative receive home health care with a home health care physician?” The questions after six months for both patients and next of kin in the interviews focused the same dimensions as the first data collection, as well as participation in health care, medical health care plan and quality of care. The patients’ interview guide focused on their own expectations and experiences. The next of kin were asked questions that reflected on both their own expectations and experiences, as well as their perceptions of their relatives’ expectations and experiences. Follow-up questions were asked to further enrich the participants’ descriptions. The majority of the patients and next of kin were interviewed in pairs, per the participants’ requests (Table 1). Separate interview guides were used, regardless of whether the participants were interviewed in pairs or alone. Field notes were written directly after the data collection had been concluded by the first author to have the researcher reflect on the initial thoughts on the interview situation and content in relation to the aim. Two pairs of participants did not participate in the follow-up data collection, and five potential participants declined participation after receiving oral information from the researchers. The number of potential participants who declined participation when informed by the contact person was not accounted for.

### Data analysis

The interviews and field notes were analyzed manually through thematic analysis using the method by Braun and Clark [76–78]. The transcripts of the interviews and field notes were read several times for familiarization and to detect patterns of meaning. Data relevant to the aim were extracted. The two data collections were separately analyzed. Initial codes were detected: nine for the first data collection and 11 for the second, in which the data from both the interviews and field notes were represented. The codes were read repeatedly; then from the data, seven initial themes were generated for the first coding and eight initial themes for the second coding. These were then reviewed through cross-checking against the two data sets as well as the field notes. The themes were then further developed and refined, which led to defining the themes in the final naming. All authors participated in this step until consensus was reached. Three main themes and eight subthemes were generated, with one main theme illustrating the expectations and two illustrating the experiences, which represented both the interviews and field notes. The first author was responsible for the initial analysis, where the coauthors contributed to the iterative analysis, where the authors detected, summarized, and interpreted the explicit and latent meaning patterns that addressed the aim. All authors had previous experience with geriatric research and three with thematic analysis. Quotes were presented in the text to illustrate the findings.

### Ethical considerations

The study was conducted according to the Declaration of Helsinki [79] and approved by the Ethical Review Authority (Dnr 2020–07149). The participants received written and oral information about the study and the voluntary nature of participation, and informed consent was obtained from all participants.

## Findings

### Expectations of safe and coherent health care

The theme represents the expectations of MICM. The participants expressed their expectations of relief when admitted to MICM, and hoped for shared responsibility in their health care matters. Furthermore, there were hopes of building a personal contact with the health care personnel (HCP) in the MICM, as well as continuity with having the same HCP responsible for the health care.

#### A hope of relief and shared responsibility

Even if the participants were recently admitted, they experienced a sense of relief in the expectations behind being admitted to MICM, and had a positive first impression of the care model. Being admitted to MICM brought the expectation of removing worry and letting go of responsibility, which was a hope for their future care. A daughter to a patient who lived alone described, *“Before, I felt like I was in a position where I had to make decisions about things I didn’t have competence for. Now, there’s these nurses, and they make the decisions instead, who knows these things.”* Having their relative admitted to MICM was something some next of kin had worked a long time for, and it was described as a relief to be able to ease part of their burden as caregiver. The sense of relief could be seen described in the field notes about one next of kin: “*The daughter handles most things about the patients’ care. She worries that the patient will fall between the cracks of health care*.” Most of participants received a direct number to the RN, which brought a sense of relief as they knew who could be expected to answer when they called. Some patients said that they had not received phone numbers for the RN and did not know how to directly contact them. It was further expected that they would no longer have to travel as much to receive primary health care. All journeys that could be prevented were described as a relief.

#### A wish for personal contact and continuity

The patients expressed a wish for continuous health care and a personalized contact with the HCP in the MICM. A patient, who had extensive experience of specialized health care prior to being admitted to MICM, said: *“What’s important is continuity, that there isn’t a bunch of different physicians. You have to start over every time there’s a new physician. I want a contact and keep it.”* Some expressed that, because of the expectation of a personal contact with the RN, the participants did not think they would need to have a contact with a physician in the same way as before admittance. Others grew worried about the possible lack of physician contact, and felt unsafe in their expectation of future care because of it. Some of the participants expressed that they did not have any expectations of MICM since they had received little or no information and not knowing who the MICM physician was made it difficult to have expectations. The lack of expectation is illustrated in the field notes: *“Neither the patient nor the next of kin had ever heard of a MICM physician before I mention it.”* Participants who had previous experience with a specific MICM physician had a sense of safety in knowing the MICM physician. The participants’ hopes for future care was for the MICM physician to check their medications and for answers about why they had become ill, even if they did not think the MICM physician could do anything about it.

### An easier daily life

At the follow-up interviews, participants described how they now had to spend less time on travel and therefore had more time to live their lives the way they wanted. The increased access to the HCP allowed for participants to let go of responsibility, and a personalized contact and the continuity provided by the MICM created a sense of safety. All of these aspects created an easier daily life for the participants.

#### Less time to travel, more time to live

The participants expressed a sense of relief the health care visits were done in the home. The relief could be seen in the field notes: *“The great distance to primary care took energy from both patient and next of kin before, which seems to be much better now.”* The participants pointed out how medication was delivered to the home instead of having to be picked up at the pharmacy, thereby making daily life easier. A patient who was living alone said, *“The best thing of it all is that I don’t have to go anywhere.”* They addressed the fact that the RN came to do exams, take bloodwork and administer treatment. It lowered the amount of times the patient needed to visit a health care facility and the number of times the next of kin had to drive them. A next of kin, an older woman living with her husband who was a MICM patient said, *“We don’t have to go anywhere, I just press the alarm, and someone helps us. It’s such a safety feeling for me… that everything isn’t on me anymore.”* However, it could be perceived as tiring to have health care in the home, and made the patients and cohabiting next of kin feel bound to the home. Still, the closeness to health care made the participants feel that, instead of having to use all their energy on traveling to a health care facility, such as the hospital, they could focus on living a good daily life. Several of the patients also received health care from other health care providers to which the next of kin had to drive them.

#### Direct access enabled reduced responsibility

The participants experienced their daily lives becoming easier after being admitted to the MICM since there was someone to help carry the burden of responsibility. Having their daily life become easier was illustrated in the field notes: *“They feel very safe with health care, and that they get the help they need quickly when they need to.”* A daughter to a patient living alone, said, *“Before, it could take half a day before you came in contact with health care, and now I just call the nurse and it’s solved.”* The RN kept track of all medical matters and there was a sense of relief in not being alone in the health care and having someone to call for assistance. Other HCP were also more accessible than they were before admittance, such as physiotherapists and occupational therapists. Some next of kin experienced that the HCP did not keep track of everything, however, meaning they could not fully let go of their responsibility to the patient.

#### A personalized contact and continuity to provide safety

For some of the participants, the expectation of personal contact and continuity was fulfilled. Having a specific RN who knew the patient’s needs made daily life easier. This was especially noticeable when the RN became sick, was on leave or ended their employment. A patient, an older woman living with her husband in the countryside said, *“The nurse is really great. When she was sick, it was like a hole in health care.”* The patients expressed that they did not have much contact with the MICM physician. Most had met the MICM physician once, if at all, which meant they had not experienced continuity in the physician’s provided care. Some expressed that they had not met the RN much either, having had several different RNs since admittance, and that it was the ANs who were the most important persons in their health care since they met them more frequently. The importance of the relationship with the AN was described in the field notes: *“The patient feels that she and the AN are like friends.”* Building a personalized contact with the RN and MICM physician was expressed as being important, and being able to talk about other things and tease each other was crucial for building a personalized contact. The participants expressed that personal characteristics influenced their sense of safety. A daughter to a patient with extensive care needs said, *“Oh god yes, it matters. Who the physician is matters!”*

### A hierarchical structure with an impact on participation

The participants experienced that the MICM had a hierarchical structure that influenced their possibility to participate in their own health care. There was a structural impact of having a singular physician, and while the home visits opened up the possibility for shared decision making, the hierarchy made the participants feel as though decisions were at times made above their heads.

#### A structural impact of having a singular physician

The participants experienced that the MICM physician’s role was to handle medical matters that the patients themselves could not handle, such as information about their medications. The MICM physicians were also said to have consulted with the RN when the patients needed emergency care. Patients experienced having had the opportunity to discuss what they wanted during the meeting with the MICM physician, but none had any memory of making a MHCP. The meetings with the MICM physician were also described as being unpersonal. A daughter to a patient living with her husband in the countryside said, *“It’s the same standard questions, and then the follow-up questions become more personal, but perhaps it doesn’t become very personal at that type of meeting with the physician.”* The patients expressed that they were not allowed to seek another opinion than the MICM physician’s, and one patient was released from MICM because she sought medical advice elsewhere. The situation was described in the field notes: *“It’s presented during the conversation that since because the patient called the primary care center to seek a second opinion, she was excluded from the MICM, but only the physician based care*.” Some patients also worried about the MICM physician having the competence to handle their complex health care needs. A patient, who had previous experience of specialized health care said, *“When you’re admitted to MICM, you can’t ask for a second opinion, and I worry about it because… what if the physician doesn’t have the competence for all my health problems? Am I allowed to call someone else?”* Participants expressed feeling safe with the MICM physician because they perceived them as being considerate and competent. Other patients expressed that their sense of safety with the MICM physician was low, as the MICM physician was quiet, could not answer questions and did not take the patients’ concerns seriously.

#### A care model with a hierarchic structure

The participants expressed noticing a hierarchic structure within the MICM, which was solidified by the participants describing themselves as being at the bottom of the hierarchy. A patient, who had extensive help from the AN and had previously met the MICM physician said, *“If we want any changes in medication, we have to go through the assistant nurse, who talks to the nurse, who talks to the physician.”* Some patients preferred this structure, since it meant they did not have to try in vain to get in touch with the HCP. Others were uncomfortable with this system. A patient, who had a close relationship with both the AN and the RN said, *“It seems that the physician is up there, and I’m down at the bottom, which feels like an old-fashioned idea.”* Some also explained that the RN and MICM physician sometimes teamed up against the patient, further cementing the hierarchy. The experience of having HCP team up against the patient was illustrated in the field notes: *“The patient gets frustrated when he speaks about how the RN acts different when it’s just the RN and the patient, as opposed to in the meeting with both the RN and the MICM physician.”* The RN and the MICM physician had a close collaboration, where they met once a week to discuss cases and made home visits together. No participant had met the MICM physician without the RN present.

#### Possible participation on the terms of the MICM structure

Patients described being a part of the dialogue about their health care in the MICM and that they received the health care they wished for and needed. The next of kin was encouraged to participate in health care in the MICM, and received information at the meeting with the MICM physician or afterwards from the RN. Some next of kin were not invited to participate nor received any information afterwards. Medical records were either kept in a locked cabinet or on digital platforms that participants could not access. Some described this as frustrating, diminishing and solidifying the hierarchy with the patient at the bottom. Some expressed that the patients were not permitted to handle their own medication, something which was described to be part of the MICM structure. A patient, who had previously handled his own medications for decades, said: *“‘What the fuck?’ I say, because they downgrade me by doing that. What do they think I do with the medications? Sell them?”*

The HCP were seen as being stressed, seen described in the field notes: *“The patient returns to pointing out that the AN is stressed several times, which he daughter seems to agree with.”* Next of kin refrained from calling the RN as a result, and it took months before the MICM physician could visit the patient. The participants viewed this as indicating that the MICM physicians did not have enough time to handle their caseload. A daughter to a patient who was living alone said, *“If the physician can’t visit for five months, she probably has too much to do.”* This was further evidenced by the MICM physician visits being postponed, cancelled or rushed, thereby further impacting participation. Others experienced that the MICM physician visit was calm and they went through the patients’ entire medical record, which made them feel as if they had the opportunity to participate in their health care.

## Discussion

In the first theme, the participants expressed an expectation of relief in the hopes of less travel, shared burden, and increased accessibility, a struggle that the participants described as having faced before admittance to the program. This struggle could be viewed as having a possible relation to what has been described as the fragmented Nordic health care system [28–30], which the MICM and the shift to local health care aimed to bridge the gap of [37, 66]. The next of kin described hopes of relief and shared responsibilities, potentially easing caregiver burden, which has been known to increase the risk of psychological stress and depression [21]. The goal of both the MICM and local health care was to provide continuity [37, 66], with the potential to meet the hopes of the participants of creating a personalized contact in their expectations.

In the second theme, the participants described the fulfilled expectation of less travel from home as being the most prominent relief. The result of less travel was in line with providing close health care according to local health care shift goals [37]. Providing health care in the home has previously been described by Swedish physicians as providing more information about the patient [67]. Receiving care within the home has been seen to have positive effects on sleep and physical activity [80], increasing patient participation, and facilitating a safe environment according to Swedish and an American study [68, 80, 81]. The positive effects could be related to how the participants experienced an easier daily life when receiving in-home health care, potentially reaching the goals of local health care [37] since the participants did not need to travel to receive at least part of their health care. Less travel may therefore be a factor in MICM contributing to meeting the local health care goal of providing person-centered care [37]. However, the patients and cohabiting next of kin also described how having health care in the home could be tiring, which was previously described in a Swedish study as a risk of disrupting the sense of home for the patient [11]. The participants in the current study did not explicitly express this, but some described a sense of being bound in place while waiting for health care visits.

In the second theme, the next of kin described how they felt at ease in their responsibility in relation to their relative’s health care but still felt that they could not fully let go of their responsibility. The MICM attempted to diminish having several health care providers [57], something the patients still described having the need for after admittance. Cross-organizational collaborations have previously been described by RN and MICM physicians as negatively influencing the quality of care, where being part of the same organization was preferred [67, 68]. Sweden is known for being a generous welfare state [28, 30]. However, the fragmentation of the Swedish health care system [29] being organized around over 300 different health care providers may make it difficult to fully provide non-fragmented health care, regardless of the quality of the local health care initiatives. The MICM has been implemented in one of 21 regions, and is therefore not a Swedish standard. The care models has been upheld as a role model in the shift to local heal care [58], but is only one of many similar initiatives to meet the goals of the local health care shift. Health care providers are struggling to meet the goals of the local health care shift [38], suggesting that, even though MICM provides local health care, individual integrated care models are not enough to ensure reaching the goals in the shift to local health care, despite the structural changes described as already being implemented [38]. Instead, it could be perceived that an even broader structural change of the whole Swedish health care system is needed to end the sense of fragmentation for HCP, patients, and next of kin.

Continuity was an expectation of the MICM [57] that involved having the same RN and MICM physician responsible for their health care during an extended period of time [66]. A few patients described having changed RN several times since admittance because the RN had quit, creating obstacles in continuity. Personal contact was described by the participants as providing a sense of security, with the participants stating that it was important that they could talk about things other than health care with their HCP. Building relationships was a goal of the shift to local health care [37], and not being able to build personal contact with the HCP was something patients in Europe had previously described as an issue [82]. In a previous study, RNs expressed that the individual differences between MICM physicians impacted the care the patients received and the RNs’ work satisfaction [68]; in addition, the participants described the individual MICM physician as crucial. Being able to build a relationship with the HCP was, therefore, seen as important to the patients and next of kin, effecting quality of care, and influencing the RNs working satisfaction.

The participants described MICM as hierarchic in the third theme, influencing the possibility of the patients and their family members participating in the patient’s care, which the MICM worked to promote [57]. The hierarchy potentially conflicted with patients being included in a partnership, an aim for person-centered care. However, the participants described having been able to talk about what they wanted during the meeting, potentially promoting participation within the MICM. The structural hierarchy in all health care organizations within Europe, with the physicians at the top and the AN at the bottom, is well known [83], and the participants placed themselves at the bottom, below the AN. Some patients preferred this structure and found comfort in it, while others struggled with it. The hierarchy of the MICM could be seen through the barriers to contacting the MICM physician, and the fact that the patient never met the MICM physician alone, with an RN always being present. On the other hand, the RN has previously described being a communication mediator between the patient and MICM physician [68]. Furthermore, the hierarchy could be seen through how the participants could not access their documentation, conflicting with the person-centered care approach of the MICM [50, 57], which could be a possible way of improving participation in the MICM in the future.

### Methodological considerations

The present study’s aim was to evaluate patients’ and next of kin’s expectations and experiences of a mobile integrated care model with a home health care physician at baseline and at six months of follow-up. The MICM physicians’ and RNs’ experiences have been described in previous publications [67, 68] which, together with the present study, are a part of a larger project studying the experiences of the MICM from different perspectives. Data collection through interviews can gain a deeper understanding of a phenomenon [84], so it was considered appropriate to use this method to address the present study’s aim. Future research may benefit from other means of data collection, something that the quantitative data collected were intended but not deemed possible to use because of the difficulties in recruiting participants. This led to a lack of opportunity to analyze the impact of the MICM from a quantitative perspective. The study’s design was done in collaboration with the municipalities, where it was deemed possible to find enough participants. However, during data collection, the contact persons expressed that the most common patient group admitted to the MICM was patients with cognitive impairments, meaning that they fell outside of the inclusion criteria.

Patients and next of kin newly admitted to the MICM were seen as relevant to the study based on the possibility of being able to capture both their expectations and experiences of the MICM, thereby strengthening the study’s credibility [85]. The participants received health care from different municipalities with different RNs and MICM physicians, allowing for a diverse view of the MICM, as opposed to the information garnered if all of the participants lived in the same municipality. Four of the municipalities that agreed to participate did not find any individuals meeting the inclusion criteria within the given time frame. The reasons for this included that the admitted patients either had cognitive impairments or needed palliative care and, therefore, were not perceived as being able to participate in a follow-up after six months. This could mean that some of the expectations and experiences surrounding specific municipalities were not incorporated into the findings. Monthly meetings were held with the contact persons, where the study and possibility for the researchers to aid the contact persons were both discussed. However, because there were contact persons responsible for recruitment, it is possible that there was selection bias to participants that the contact person thought might be talkative or thought would speak favorably about the MICM. Furthermore, patients choosing the next of kin to participate might have impacted the findings as well. The decision to meet the participants for follow-up after 6 months was made after a discussion with health care providers who claimed that patients should have met the MICM physician at least once during that time period. Some patients did not, however, which may be related to the strain that the COVID-19 pandemic has had on health care, as well as the lack of time that the participants in this, as well as the previous, studies [67, 68] have described. The result of this could be that a few patients did not have experience of receiving care from the MICM physician, hence struggling to relate experiences surrounding the physician care in the follow-up interview. The participants did, however, have extensive experience in the rest of MICM, so it was not deemed as influencing the findings.

The present study’s credibility is strengthened by the method of data collection, the description of the analysis process, and the presentation of representative quotes in the findings. Dependability was enhanced through iterative discussion among the authors of the manuscript. The transferability is up to the reader through the thorough description of participants and data collection and analysis process [85].

## Conclusion

The participants described how they, at admittance to the MICM, had an expectation of receiving safe and coherent health care that they hoped would create a sense of relief, as well as being able to share responsibility. Furthermore, they wished for personal contact with the MICM physician and RN and to have the same HCP visit them to build relationships. However, some experienced having received little or no information about MICM. The lack of information could be seen as threat to person-centered care since it hinders the possibility to have equal part in one’s own health care. After six months, the participants expressed that the MICM had provided an easier daily life, where the health care they received in the home meant they had to spend less time traveling to different health care appointments and, instead, could use their time and energy to focus on what was important in their lives. These findings could be related to how older persons and their next of kin prefer home health care because of how it lowers travel time and saves energy, meeting the goal of the shift to local health care. Being able to reduce travel was upheld as the most prominent relief. The direct access to HCP reduced their responsibility, even if it did not fully remove it. Some had received a direct number to the RN, while other had not, where direct access to a RN could be seen as possibility to improve the MICM in the future. The participants described it as important to create personalized contact with the HCP and that the individual HCP mattered to them. The MICM was described as a hierarchic structure that impacted participation, here as understood through having a singular physician responsible for their medical care. Furthermore, the participants experienced the structure of the MICM as negatively influencing their participation because they saw themselves as being the lowest in the hierarchy. This stands in contrast to the person-centered base of the MICM and local health care, of creating a partnership between the patient and HCP, which would be of value to explore in the future. However, the participants were mainly positive toward the MICM and had experiences of the MICM increasing participation. The MICM could be understood as improving several of the systematic problems that can be found in the perceived fragmented health care system and as being a possible stepping stone toward the shift to local health care. While the MICM provides the organizational prerequisites for person-centered care, it seemed to lack the interprofessional and relational focus. To structurally focus on interprofessional and patient professional relationships is a possible way to develop the MICM, and other integrated care models, in the future. Furthermore, it could be perceived that a structural change of the whole Swedish health care system is needed to diminish the sense of fragmentation for HCP, patients, and next of kin.

## Data Availability

The data sets analyzed during the current study are not publicly available due to ethical principles and the guidelines of the Swedish Ethical Review Authority, but data not comprising confidential information are available from the corresponding author on reasonable request.

## Abbreviations

RN: Registered nurse
MICM physician: Home health care physician
AN: Assistant nurse
HCP: Health care professionals (registered nurses, home health care physicians, assistant nurses working in health care, occupational therapists, and physical therapists)
MHCP: Medical health care plan
